# Clinical prognosis and related molecular features of hepatitis B-associated adolescent and young adult hepatocellular carcinoma

**DOI:** 10.1186/s40246-023-00500-9

**Published:** 2023-06-13

**Authors:** Tao Lv, Bo Zhang, Xi Xu, Chenhao Jiang, Daofeng Zheng, Diao He, Yongjie Zhou, Jiayin Yang

**Affiliations:** 1 Department of Liver Transplant Center, Transplant Center & Lab of Liver Transplantation, West China Hospital of Sichuan University, Sichuan University, Chengdu, 610041 China; 2 Department of Critical Care Medicine, West China Hospital of Sichuan University, Sichuan University, Chengdu, 610041 China; 3 Key Laboratory of Transplant Engineering and Immunology, NHC, West China Hospital of Sichuan University, Sichuan University, Chengdu, 610041 China

**Keywords:** AYA HCC, Whole transcriptome sequencing, MPIs, Fatty acid metabolism, ceRNA

## Abstract

**Background:**

Inattention has been given to the pathogenesis of adolescent and young adult (AYA) hepatocellular carcinoma (HCC). Due to the more advanced tumor progression and poorer prognosis of AYA-HCC, together with a better tolerance ability, noncirrhotic background, and a stronger willingness to treat AYA-HCC, clinical and molecular biology studies are urgent and necessary, especially for those with hepatitis B infection.

**Methods:**

For clinical aspects, the overall survival, the recurrence-free survival, and the Cox analyses were performed. Then, functional analysis, gene clustering, metabolic-related analysis, immune infiltration and competing endogenous RNA (ceRNA) construction were carried out using whole transcriptome sequencing technique.

**Results:**

Based on the clinical information of our HCC cohort, the overall survival and recurrence-free survival rates were worse in the AYA group than in the elderly group as previously described. According to our whole transcriptome sequencing results, functional analysis revealed that metabolism-related pathways as well as protein translation and endoplasmic reticulum processing were enriched. Then the hub metabolism-related genes were screened by metabolite–protein interactions (MPIs) and protein–protein interactions (PPIs). Fatty acid metabolism is a crucial component of metabolic pathways, abnormalities of which may be the reason for the worse prognosis of HBV-AYA HCC. Finally, the relationship of disrupted expression of metabolism-related genes with immune infiltration was also analyzed, and the lncRNA‒miRNA‒mRNA-related ceRNA network for HBV-AYA HCC was constructed, which may provide new cues for HBV-AHA HCC prevention.

**Conclusion:**

The worse prognosis and recurrence rate of HBV-AYA HCC may be related to abnormalities in metabolism-related pathways, especially disorders of fatty acid metabolism.

**Supplementary Information:**

The online version contains supplementary material available at 10.1186/s40246-023-00500-9.

## Background

Hepatocellular carcinoma (HCC), known as one of the most common abdominal malignancies, ranks sixth in incidence and fourth in mortality worldwide, and approximately 50% of newly diagnosed HCC patients are from China, 50 ~ 80% of whom are associated with HBV infection. According to the National Comprehensive Cancer Network (NCCN) guidelines, the adolescent and young adult (AYA) HCC patients are defined as patients between the ages of 15 and 40 with HCC [1]. Although the proportion of AYA HCC patients is relatively small, due to the large base of HCC patients in China and the existence of HBV-related problems such as mother-to-child transmission, AYA HCC patients, especially HBV-related AYA HCC patients cannot be ignored.

Generally, compared with elderly patients, AYA HCC patients have faster tumor progression, later tumor stage, and more malignant pathological types. Therefore, the prognosis of AYA HCC patients is often worse than that of elderly patients. It is mainly reflected in the long-term overall survival rate and recurrence-free survival rate of AYA HCC patients, which are worse than those of elderly patients [2–6]. However, due to physical, psychological, and social factors, AYA HCC patients have better physical function and can endure more intensive treatment such as liver resection, TACE and targeted therapy than elderly patients. In addition, AYA HCC patients tend to be more willing to undergo treatment and choose more active treatment strategies [7, 8].

For a long time, due to the low proportion of HBV-AYA HCC patients, attention has seldom been paid to the molecular and genetic aspects of HBV-AYA HCC. Relevant literature has reported that the expression of AR, MRP1, MGMT, SPARC and other proteins in AYA HCC patients is significantly lower than that in the middle-aged and elderly groups, and the activation of the PI3K/Akt/mTOR and Wnt/β-catenin pathways is also weaker. Further genome-wide association studies indicated that mutations in *TP53*, *CTNNB1*, and *PTEN* are less common in AYA HCC patients [9, 10]. However, the molecular features of AYA HCC, especially HBV-AYA HCC, remain largely unknown.

In this study, we systematically explored the differences in long-term clinical prognosis between HBV-AYA and elderly HBV-HCC patients in our center and comprehensively analyzed the molecular features of HBV-AYA HCC by whole transcriptome sequencing. Meanwhile, combined with the data of TCGA database, we also analyzed the hub genes and pathways that have a significant impact on prognosis, which may provide new insights and cues for HBV-AHA HCC pathogenesis and prevention.

## Materials and methods

### Clinical study design

All HCC patients included in this study were HBV infected, and finally 289 HBV-related AYA HCC patients (15–40 years old) and 257 HBV-related elderly HCC patients (> 60 years old) who underwent a hepatectomy in West China Hospital, Sichuan University, Chengdu, China from 2010 to 2014, were included. The data of all patients were retrospectively reviewed. The inclusion and exclusion criteria, laboratory and histopathological examinations, and follow-up plans were listed in Additional file 1. Overall survival (OS) and recurrence-free survival (RFS) were the endpoints of our study. OS was calculated from the date of radical liver resection to the date of patient death or the date of the last follow-up visit. DFS was measured from the date of radical liver resection to the date when tumor recurrence was diagnosed.

### Collection of HCC specimens and sequencing

Fresh surgical specimens of newly diagnosed liver cancer patients were collected in our hospital between January 2020 and January 2021. Ten pairs of HCC specimens and corresponding adjacent tissues from HBV-AYA HCC patients and 10 pairs of HCC and adjacent tissues from elderly HBV-HCC patients were randomly selected for whole transcriptome sequencing, and their corresponding clinical information of them is listed in Additional file 2: Table S1. All specimens were obtained with the informed consent of the patients, and this study also passed the ethical approval of the hospital. The sequencing work of this study was completed by LC-Bio Technologies (Hangzhou) Co., Ltd.

### Whole transcriptome sequencing of AYA HCC

Differential expression analysis of mRNAs, lncRNAs and miRNAs in the AYA group and elderly group was performed, respectively. The AYA unique differential RNAs were analyzed by Venn diagram. Heatmaps and volcano plots were plotted by online website tool (https://www.bioinformatics.com.cn).

For the differentially expressed RNAs in AYA HCC, GO and KEGG functional analysis, gene set enrichment analysis (GSEA) [11] and age-related GSEA were performed. According to the corresponding age of the HBV-HCC patients included for sequencing, Mfuzz clustering analysis was performed and the genes were set into 5 groups. Furthermore, weighted correlation network analysis (WGCNA) [12] was carried out combining differential gene expression of tumor tissues and clinical indicators. Protein‒protein interaction (PPI) network [13] was established by STRING and the metabolite‒protein interaction (MPI) network was constructed as previously described [14]. The metabolism-related hub genes were screened out from the intersection of the key genes of two networks. Finally, the lncRNA‒mRNA coexpression relationship and the differential miRNA‒mRNA and the differential miRNA‒lncRNA regulatory relationships were integrated to construct the competitive endogenous RNA (ceRNA) network [15]. Centerd on downregulated lipid metabolism-related miRNAs in the ceRNAs, partial RNAs were verified by RT‒qPCR (Additional file 4: Table S3).

### TCGA data processing and analysis

Gene transcriptome data of liver hepatocellular carcinoma from TCGA-LIHC provided by the University of California Santa Cruz (UCSC) Xena database [16] were used in this study for further validation. Using principal component analysis (PCA), samples were divided into two groups with PC1 = PC2 as the dividing line. Differential gene analysis and functional enrichment was also performed. PPIs of key pathways were established with the outcomes of GSEA. The Kaplan–Meier method was used for prognosis analysis. Moreover, immune infiltrate and tumor microenvironment (TME) analyses were performed using three methods: ESTIMATE [17], CIBERSORT [18] and 29 functional gene expression signatures (Fges) proposed by Bagaev et al. [19].

### Statistical analysis

SPSS 25.0 (IBM, New York, USA), R (version 4.1.2), Microsoft Excel and GraphPad Prism 7 software were used for statistical analysis in this study. The two-tailed Welch corrected unpaired t test was used for continuous variables that conformed to the normal distribution, and the Wilcoxon rank sum test was used for continuous variables that did not conform to the normal distribution. Dichotomous variables were compared using the chi-square test or Fisher's exact test, and rank variables were compared using the Mann‒Whitney U rank-sum test. Survival curves were drawn using the Kaplan‒Meier method, and differences between the two groups were compared by the log-rank test. A *p* value < 0.05 was considered statistically significant.

For more details, see the Additional file 1.

## Result

### Clinicopathologic characteristics and survival outcomes

**Fig. 1:**
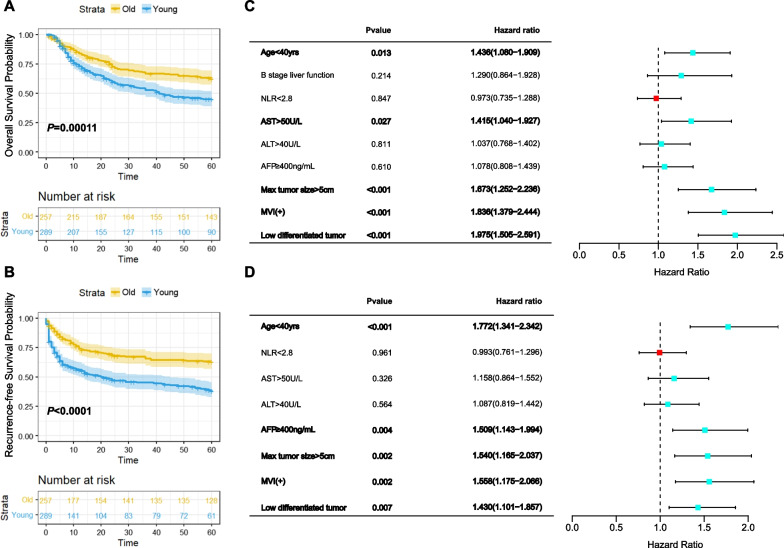
Retrospective analysis of the medium- and long-term prognosis and recurrence of HBV-AYA and elderly HBV-HCC patients.** A** comparison of overall survival rates with number at risk between the two groups;** B** comparison of recurrence-free survival rates with number at risk between the two groups; **C** multivariate Cox regression analysis for overall survival; **D** multivariate Cox regression analysis for recurrence-free survival. P values and hazard ratios (HR) with 95% confidence intervals (CIs) are shown in the left table. The right part is the forest plot of HR

According to the criteria, 289 AYA patients and 257 elderly patients were finally involved in this study. The characteristics of all the patients are listed in Additional file 3: Table S2. Among the preoperative indicators, except for the haemoglobin count, platelet count, alanine aminotransferase, albumin, and AFP, there was no significant difference. In pathological indices, only MVI had a significant difference. The median OS time of the AYA group was 41 months, while the median survival time of the elderly group did not reach the midpoint. The 1-, 3-, and 5-year OS rates of the AYA group were 72.9%, 52.8%, and 44.9%, respectively, which were significantly lower than those of the elderly group (83.8%, 66.6%, and 62.3%, respectively, *P* < 0.01) (Fig. 1A). Moreover, the RFS of the two groups showed a more significant difference, with 56.1%, 45.2%, and 38.0% of 1-, 3-, and 5- year RFS in the young group and 73.8%, 65.5%, and 63.1% of 1-, 3-, and 5- year RFS in the elderly group. The median RFS time of AYA HCC patients was only 20 months (Fig. 1B).

All significant factors in the univariate analysis and other clinically meaningful data, such as age, AFP and MVI, were entered into the multivariate analysis, which was expressed as hazard ratio (HR) and 95% confidence interval (CI). Age < 40 years, abnormal AST, max tumor size > 5 cm, positive MVI and low differentiated tumor were the main impacts of OS (Fig. 1C), while age < 40 years, AFP ≥ 400 ng/mL, max tumor size > 5 cm, positive MVI and low differentiated tumor were the major contributing factors of RFS (Fig. 1D). Similar to previous studies, we found that OS and RFS of the AYA group were worse than those of the elderly group, and youth was an independent risk factor for both.

### Metabolic pathways were critical for AYA HCC

**Fig. 2:**
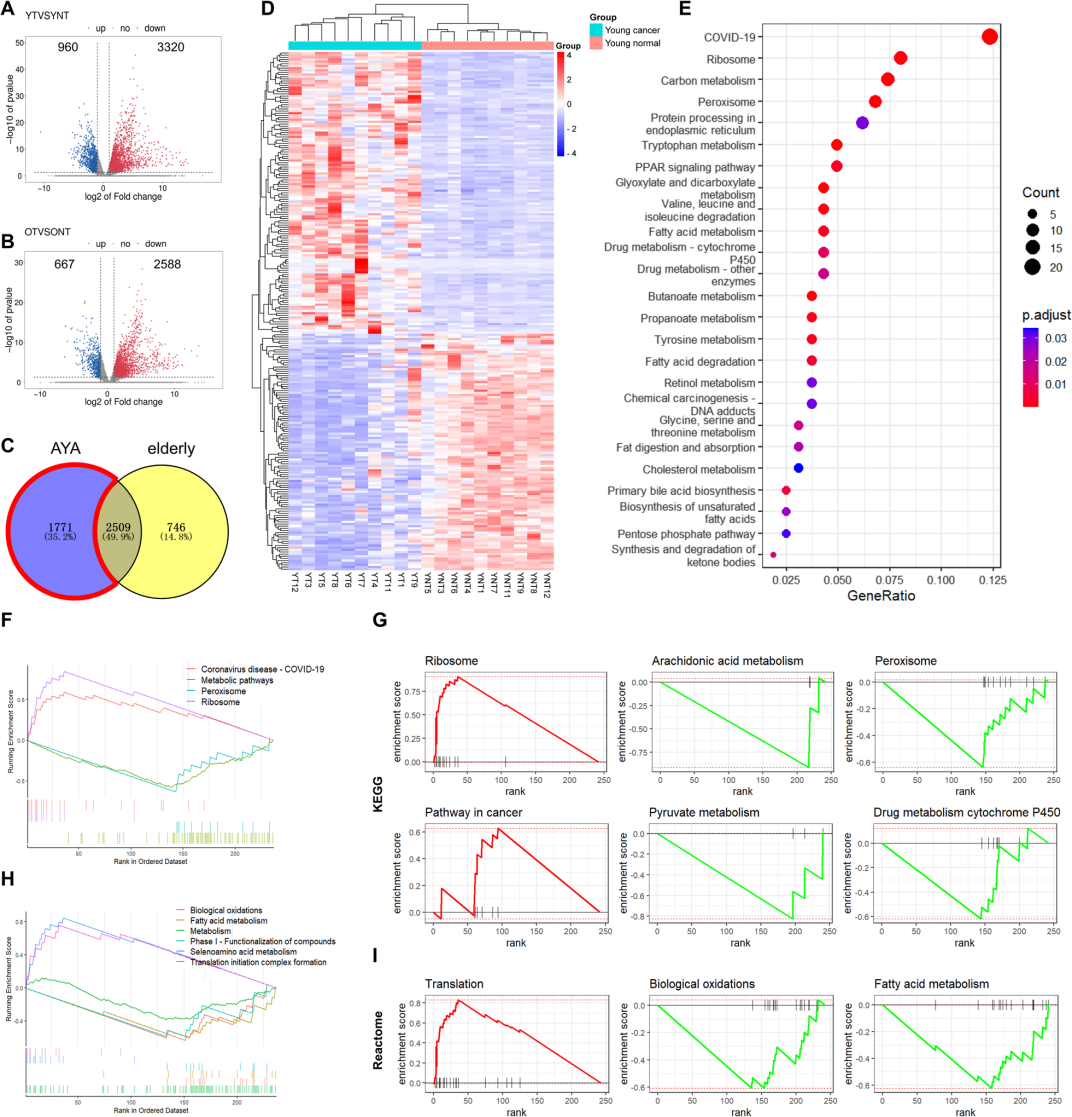
Unique differentially expressed mRNAs of HBV-AYA HCC patients and the functional analysis. **A**, **B** volcano plot of differentially expressed mRNAs in the AYA group (**A**) and the elderly group (**B**);** C** venn diagram of differentially expressed mRNAs between the AYA and elderly groups. The part in the red box is the young unique differentially expressed genes; **D** heatmap of AYA unique differentially expressed mRNAs; **E** bubble plot of enriched pathways of the differentially expressed genes; **F, H** GSEA of all genes in the AYA group. The top plot is based on the KEGG database (**F**), and the bottom plot is based on the Reactome database (**H**); **G, I** age-related GSEA analysis of 241 genes. The above 6 are based on KEGG (**G**), and the rest are based on Reactome (**I**). The *p* value of the pathway was < 0.05. YT, tumor sample of the AYA group; YNT, paratumor sample of the AYA group; OT, tumor sample of the elderly group; ONT, paratumor sample of the elderly group; p.adjust, tested by hypergeometric distribution, adjusted by the Benjamini and Hochberg method

To explore the HBV-AYA HCC-specific molecular features, whole transcriptome sequencing was carried out. First, differential mRNAs in tumor and adjacent tissues were sorted out in the AYA group and the elderly group, respectively (Fig. 2A and B). There were 4280 differentially expressed mRNAs in the AYA group and 3255 differentially expressed mRNAs in the elderly group, among which there were 1771 unique differentially expressed mRNAs in the AYA group (Fig. 2C). Considering that the role of mRNAs with extremely low expression is negligible to some extent, we further set the screening criteria as corrected p value < 0.05 and FPKM > 15, and 241 unique differentially expressed mRNAs were found in HBV-related AYA HCC patients (Fig. 2D).

KEGG functional analysis of unique differentially expressed AYA genes revealed many metabolic pathways, such as lipid metabolism, carbon metabolism, amino acid metabolism, and the PPAR signalling pathway. In addition, it is also enriched in ribosomes, protein processing in endoplasmic reticulum (ER), etc. Additionally, GSEA enriched similar pathways related to metabolism and protein translation (Fig. 2E and F). The age-related GSEA revealed that metabolism-related pathways and peroxisomes were enriched in the KEGG database, which showed a clear downwards trend (Fig. 2G). For the Reactome database, the downregulated genes were enriched in fatty acid metabolism and biological oxidation, while the upregulated genes were enriched in protein translation-related pathways (Fig. 2I). These results were consistent with the previous enrichment analysis results, both of which inferred that HBV-AYA HCC development may have a strong association with metabolic levels.

### Mfuzz clustering analysis divided differential genes into five clusters

**Fig. 3:**
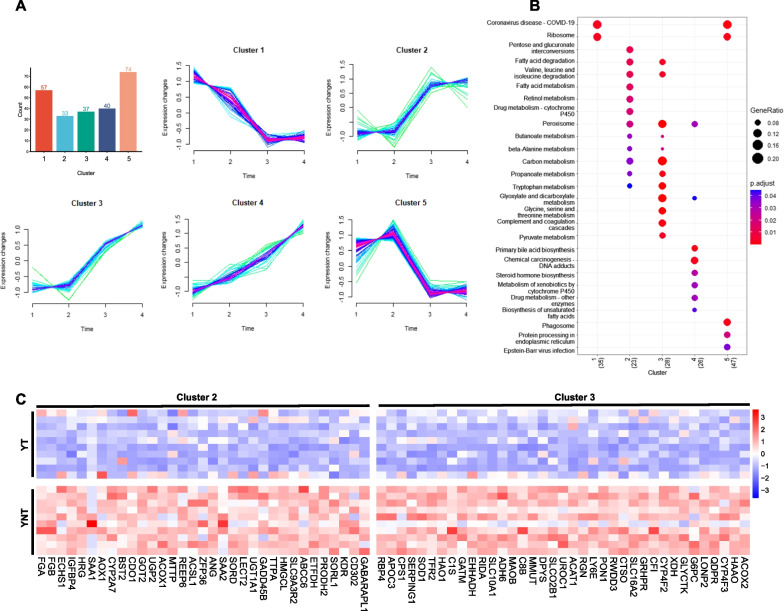
Mfuzz fuzzy cluster analysis of 241 genes. **A** This plot is a Mfuzz timing analysis clustering chart. For each cluster, the abscissa is the age grouping from youth to old age and the ordinate coordinate is the expression of genes. Each polyline represents the expression change of a gene at different ages; **B** KEGG pathway enrichment bubble plot of these 5 clusters; **C** heatmap of all differentially expressed genes in Clusters 2 and 3 in HBV-AYA HCC patients. YT, tumor sample of the AYA group; YNT, paratumor sample of the AYA group

To further analyse the functions of differentially expressed genes, and deeply understand how the expression of these genes changes with age, the trend of expression of genes according to different age groups was divided into five clusters using Mfuzz analysis. The number of genes in Clusters 1 to 5 was 57, 33, 37, 40, and 74, respectively (Fig. 3A). Clusters 1 and 5 were genes highly expressed in HBV-AYA HCC patient tissues but decreased in the elderly group. Importantly, the expression of genes in Cluster 1 showed a downwards trend with increasing age in the AYA group. Clusters 2, 3, and 4 were genes with low expression in the AYA group and relatively high expression in the elderly group.

The genes in Clusters 1 and 5 were mainly enriched on ribosomes, and the genes in Cluster 5 were also associated with phagosomes and endoplasmic reticulum. The genes in Cluster 2 were mainly enriched in fatty acid metabolism and other metabolic pathways, while those in Cluster 3 were more concentrated in carbon metabolism and protein-related metabolism in addition to lipid metabolism (Fig. 3B). The detailed genes in Cluster 2 and 3 are displayed in a heatmap (Fig. 3C).

### The coexpression of some of upregulated genes was correlated with age

**Fig. 4:**
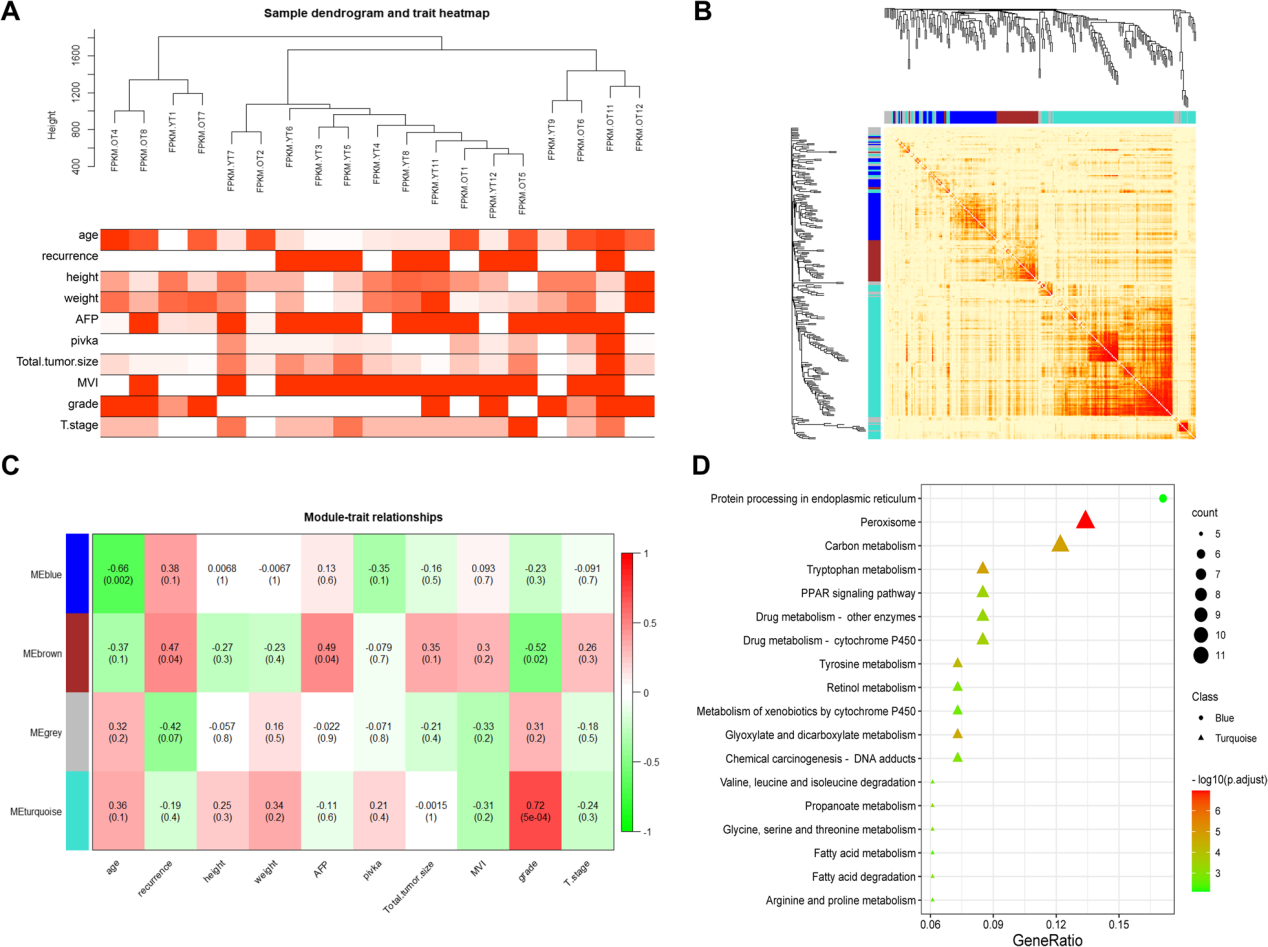
WGCNA analysis of young unique differentially expressed genes. **A** The upper part is the dendrogram of tumor samples. The heatmap shows the clinical phenotypes of the samples, and a redder box indicates a greater numeric value or rank. MVI and recurrence within one year are binary variables; **B** network heatmap plot of AYA unique differentially expressed genes. The different colors at the bottom of the clustering tree represent different modules; **C** heatmap of module-clinical phenotype relationships. The numbers in each cell are the correlation coefficient and p value (in parentheses); **D** the pathway enrichment plot of genes in blue (circle) and turquoise (triangle) modules. AFP, alpha-fetoprotein; MVI, microvascular invasion

To further explore the age-related genes, the WGCNA algorithm was used to perform the gene expression correlation analysis on 20 liver tumor samples. It was found that the No. 9 sample in the elderly group was obviously an outlier, so it was eliminated (Additional file 2: Table S1). The remaining 19 samples were included for further analysis, and the sample correlation dendrogram and heatmap of clinical phenotypes were drawn (Fig. 4A). These differentially expressed genes were divided into 3 modules, namely, the blue module, brown module, and turquoise module, and the coexpressed gene network heatmap of the differentially expressed genes is shown in Fig. 4B.

WGCNA defined the data of different modules as eigengenes. By calculating the correlation coefficient between eigengene and clinical phenotype data, the correlation diagram between modules and phenotype was obtained. As shown in Fig. 4C, the blue module had a significant negative correlation with age (*P* = 0.002), which showed that with increasing age, the expression of these genes in tumors was downregulated. In addition, the turquoise module and age had a certain positive correlation, but no significant difference was reached. Intriguingly, these genes had a strong positive correlation with the grade of pathological differentiation (*P* < 0.001): that is, the lower the gene expression, the worse the pathological grade (Fig. 4C). Functional analysis of the blue module gene showed that it was mainly concentrated in the pathway of protein processing in the ER. Moreover, the functional analysis of the turquoise group showed that these downregulated genes were mainly related to peroxisomes, carbon metabolism, amino acid metabolism, lipid metabolism, and the PPAR signalling pathway (Fig. 4D). In contrast, the grey module was a type of gene set that cannot be divided into any module, and the coexpression trend of genes in grey module was not obvious.

### Low expression of five-hub metabolism-related genes led to worse prognosis of HCC

**Fig. 5:**
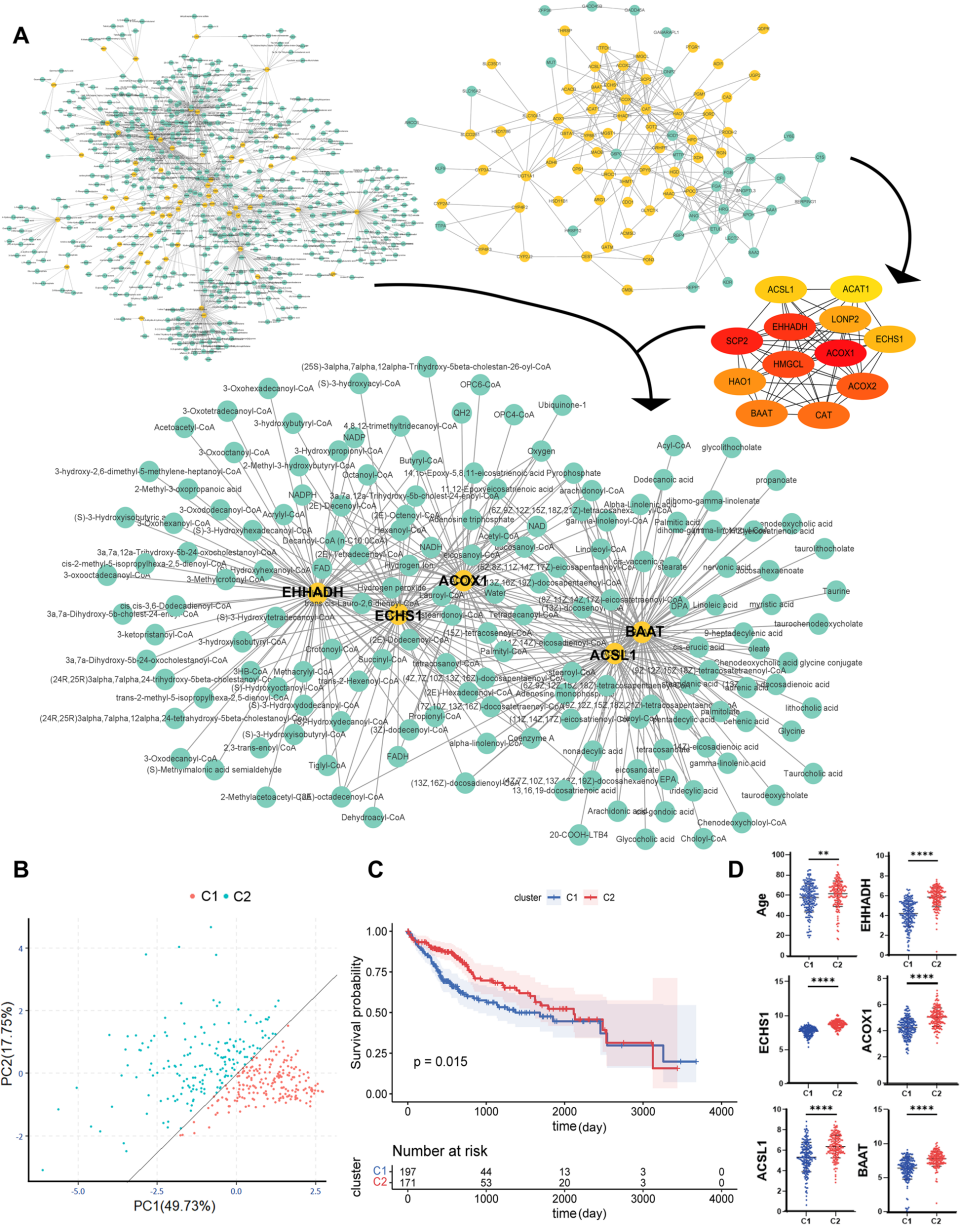
MPI network construction of metabolism-related genes and identification of two TCGA HCC subtypes. **A** The network in the upper left corner is the MPI network of AYA unique differential metabolism-related genes, the yellow dot is the gene, and the green dot is the metabolite. The network in the upper right corner is the PPI network of differentially downregulated genes, with the yellow dot being a metabolism-related gene and the green dot being another downregulated gene, below which 12 hub genes were screened by the MCC algorithm. The network below is the MPI network of 5 hub metabolism-related genes; **B** PCA of TCGA-HCC tumor samples based on 5 hub genes. Points are colored according to the consensus clustering results, and the two subtypes can be exactly separated by the line PC1 = PC2; **C** Kaplan–Meier plot of the differential prognosis between the two subtypes; **D** Significant differences between the two subtypes in the age and 5 hub genes. *p < 0.05, **p < 0.01, ***p < 0.001, ****p < 0.0001 and ns for not significant

Although Mfuzz and WGCNA proved that the unique AYA HCC genes were related to metabolic pathways, we were unable to judge which genes were more critical. Sixty-four metabolism-related genes among the downregulated differentially expressed genes were screened according to the metabolism pathways in the KEGG and Reactome databases. Combining the metabolism-related products in databases such as KEGG, Reactome, Human-GEM, and BRENDA, a metabolite–protein interaction (MPI) network was constructed (Fig. 5A), in which there were 1118 protein-metabolite pairs in the network, including 531 metabolites and 63 metabolism-related genes. In addition, based on the downregulated genes, a PPI network was constructed. The top 12 key genes were obtained by using the MCC algorithm in cytoHubba [20], including *ACOX1*, *SCP2*, *EHHADH*, *HMGCL*, *ACOX2*, *CAT*, *BAAT*, *HAO1*, *LONP2*, *ECHS1*, *ACSL1* and *ACAT1*. The metabolism-related genes in the MPIs were sorted by degree in CytoNCA, and the top 10 genes were *BAAT*, *EHHADH*, *ACSL1*, *CYP3A7*, *UGT1A1*, *ECHS1*, *ACOX1*, *GSTA1*, *MGST1* and *MAOB*. Taking the intersection of the two networks, five key metabolism-related genes were finally identified, namely, *EHHADH*, *ECHS1*, *ACOX1*, *ACSL1* and *BAAT* (Fig. 5A). The functions of the five genes were mainly concentrated in lipid metabolism, especially fatty acid degradation, unsaturated fatty acid biosynthesis and peroxisome.

To further explore the relationship between this metabolic abnormality and the prognosis of patients with HCC, the PCA of TCGA liver cancer samples based on the expression of 5 key genes was performed. All TCGA tumor samples were neglected the infection status of HBV and divided into two groups (Fig. 5B). Combined with the gene expression and clinical data of the two groups, the OS curve based on the C1 and C2 groups, as well as the difference analysis between the groups on the age distribution and the expression levels of 5 hub metabolism-related genes were drawn (Fig. 5C, D). The OS rate of C1 was significantly lower than that of C2 (*P* = 0.015). There were significant differences in the age distribution and the expression of five hub genes between the two groups (*P* < 0.01). Intriguingly, the age distribution of the C1 group was relatively younger and the expression of five key genes was lower. Therefore, possible speculations could suggest that abnormal lipid metabolism had a critical impact on the pathogenesis and prognosis of HCC. Additionally, when the age distribution was younger, the expression of lipid metabolism-related genes was relatively lower, which was in line with the results obtained from our sequencing data.

### Functional analysis and immune infiltration landscape of two subtypes of HCC

**Fig. 6:**
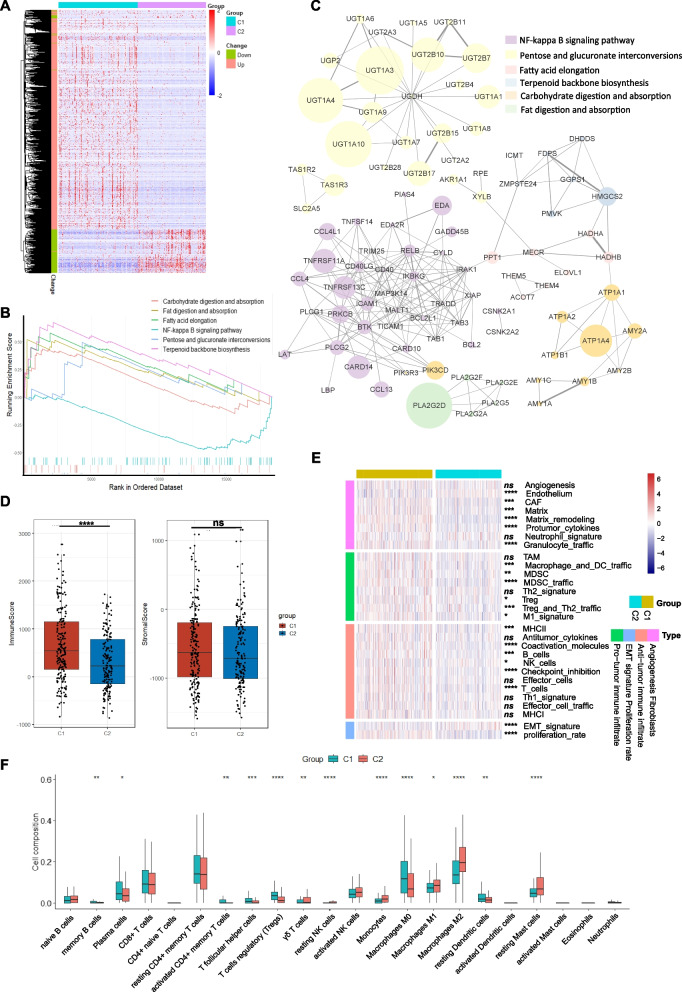
Differential analysis, functional enrichment analysis and immune infiltration landscape of the two subtypes.** A** Heatmap of 1285 differentially expressed genes between the C1 and C2 group; **B** GSEA based on the KEGG database; **C** PPI network of differentially expressed genes related to the above pathways; **D** Box and dot plot showing the stromal score and immune score by ESTIMATE; **E** heatmap of the enrichment score of 29 Fges in HCC patients from the C1 and C2 groups. The significance analysis of each item is displayed on the right side of the heatmap;** F** Boxplot showing the proportion of the 22 types of immune cells in HCC patients from the two subgroups by CIBERSORT. *p < 0.05, **p < 0.01, ***p < 0.001, ****p < 0.0001 and ns for not significant

Differential gene analysis was performed on the two groups of tumor samples, and a total of 1285 differentially expressed mRNAs were screened with |log2-fold change (FC)|> 2 and a corrected *p* value < 0.01 as the threshold (Fig. 6A). GSEA based on C1 and C2 was performed on all mRNAs according to the fold changes between the two groups. Compared with C2, C1 was enriched and downregulated in metabolism-related pathways, such as fatty acid elongation, fat and carbohydrate digestion and absorption. Conversely, the NF-κB pathway was enriched with many upregulated genes in C1 (Fig. 6B). The PPI network was constructed for the differentially expressed genes enriched in the above pathways (Fig. 6C). Among the downregulated pathways in C1, the fatty acid elongation pathway was at the core of these pathways and highly associated with other pathways except the fat digestion and absorption pathway, including genes such as *PPT1*, *MECR*, *HADHA*, *HADHB*, *ELOVL1*, *ACOT7*, *THEM4*, and *THEM5*. The differentially expressed genes of the NF-κB pathway were related to TNF receptors and apoptosis. *ICAM1*, *PLCG1*, *PLCG2* and other genes in the pathway are also related to tumor migration [21].

The tumor microenvironment (TME) consists of a variety of nontumor cells, including immune cells and stromal cells, which profoundly impact cancer growth and invasion [22]. By ESTIMATE analysis, we noticed that the stromal score showed no difference between the C1 and C2 groups. However, the immune score of C1 was significantly higher than that of C2 (Fig. 6D). The above analyses indicated that more pronouncedly abnormal fatty acid metabolism in HCC might have a more profound impact on components of the TME. The cellular and functional TME properties can be reflected by the expression of 29 functional gene signatures (Fges) described by Bagaev et al. [19] Based on their description, the patients in the C1 group were characterized by the elevated expression of Fges associated with cancer-associated fibroblast (CAF) activation, protumor immune infiltration, myeloid-derived suppressor cells (MDSC), Treg, M1 signature, and a higher proliferation rate. Some components of antitumor immune infiltrates, such as MHCII, coactivation molecules, B cells, T cells and checkpoint inhibition showed a significantly higher enrichment in the C1, whereas no difference was observed in the remaining antitumor immune infiltrate-related terms, such as antitumor cytokines (Fig. 6E). We conducted the following analysis by using CIBERSORT, which showed that memory B cells, activated CD4 + memory T cells, T follicular helper cells, Tregs, M0 macrophages, resting dendritic cells were significantly upregulated in the C1, while γδ T cells, resting NK cells, monocytes, M2 macrophages, and resting mast cells were significantly decreased (Fig. 6F). There was no significant difference in CD8 + T cells, CD4 + naive T cells or naive B cells. Although there were increases and decreases in different types of macrophages, M2 macrophages with antitumor function increased more significantly in the C2, and the overall amount of macrophages, dendritic cells, and the M1 signature were increased in the C1 with no difference in tumor-associated macrophages (Fig. 6E, F). In general, the upregulation of antitumor immune infiltration was not as significant as that of the protumor components.

### ceRNA network construction for HBV-AYA HCC

**Fig. 7:**
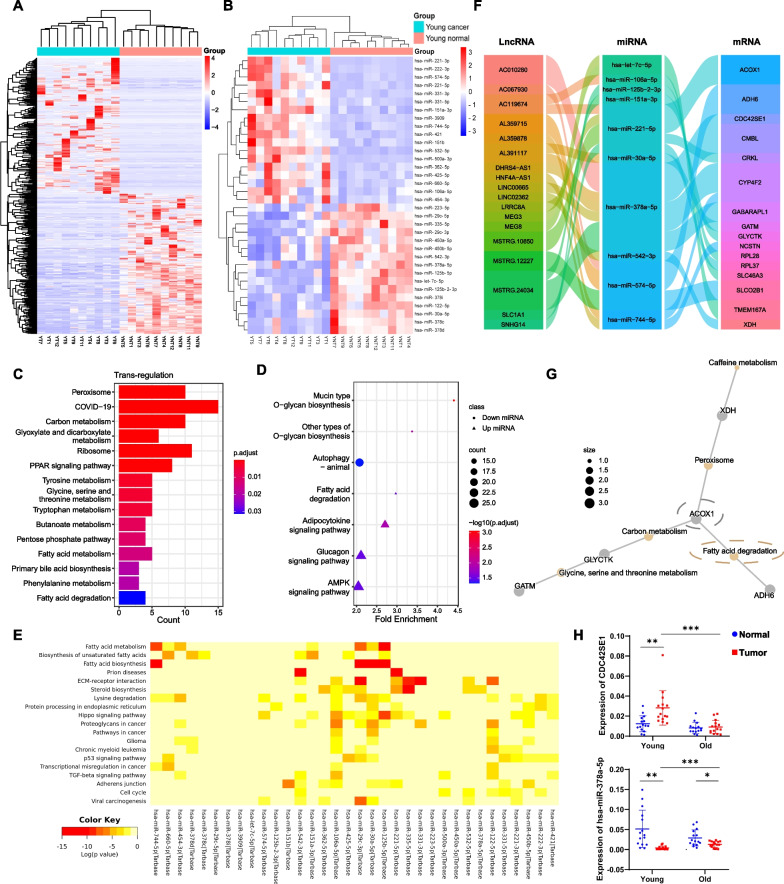
ceRNA network establishment of HBV-AYA HCC. **A** Heatmap of AYA unique differentially expressed lncRNAs; **B** heatmap of AYA unique differentially expressed miRNAs; **C** KEGG functional analysis results of target genes under trans-regulation of lncRNAs. **D** the KEGG enrichment bubble diagram of miRNA target differentially expressed genes; **E** KEGG pathway prediction of differentially expressed miRNAs based on the TarBase database; **F** the lncRNA‒miRNA‒mRNA ceRNA network constructed with the differentially expressed lncRNAs, miRNAs and mRNAs. The line between the two columns indicates the existence of a regulatory relationship, and the longitudinal width of each RNA frame represents its importance in the network; **G** the relationship network between the enriched KEGG pathways and the corresponding differential genes. The yellow dot is the pathway, the grey dot is the gene, and the size of the yellow dot represents the number of genes enriched; **H** the RT‒qPCR results of *CDC42SE1* and hsa-miR-378a-5p. *p < 0.05, **p < 0.01 and ns for not significant

To further explore the molecular features of AYA HCC, we also screened AYA unique differentially expressed lncRNAs and miRNAs. Through preliminary screening, 1504 differentially expressed lncRNAs in the AYA group and 1103 differentially expressed lncRNAs in the elderly group were obtained, of which 982 differentially expressed lncRNAs were unique to the HBV-AYA patients (Additional file 5: Fig. S1A–C). After further defining the adjusted adjust *p* value < 0.05, a total of 445 AYA unique differentially expressed lncRNAs were obtained (Fig. 7A). According to the condition of |log2-fold change (FC)|> 1 and *p* value < 0.01, 134 differentially expressed miRNAs were screened out in the AYA group, and 88 differentially expressed miRNAs were screened out in the elderly group (Additional file 5: Fig. S1D–F). Finally, 34 AYA unique differentially expressed miRNAs were screened, of which 18 were upregulated and 16 were downregulated in tumor tissue (Fig. 7B).

Furthermore, we calculated the correlation coefficient between differential lncRNAs and differential mRNAs to roughly predict the function of target genes that were trans-regulated by these lncRNAs. We set the threshold as the correlation coefficient > 0.85 and p value < 0.05, and 144 differential target mRNAs were obtained. It could be found that there are two major types of functions that were enriched, one is the ribosome-related genes, and the other is related to various metabolic pathways including fatty acid metabolism (Fig. 7C). Target mRNAs of miRNAs were screened by TargetScan and miRanda, and a total of 4305 differential miRNA‒mRNA pairs were obtained. Functional analysis of target genes was performed, respectively, in the upregulated and downregulated differentially expressed miRNA groups. The upregulated group was mainly enriched in fatty acid degradation, adipocytokine and the AMPK signalling pathway (Fig. 7D). The AMPK signalling pathway is closely related to cell nutritional status, glucose metabolism and lipid metabolism [23]. Meanwhile, taking the advantage of the mirPath website and the experimental TarBase database, 34 differentially expressed miRNAs were summarized by KEGG. It was found that fatty acid metabolism, biosynthesis of unsaturated fatty acids, fatty acid biosynthesis, ECM-receptor interaction, steroid biosynthesis, and the Hippo signalling pathway were enriched (Fig. 7E).

First, miRNAs with a *p* value < 0.05 in the elderly group were excluded. Then combined with the KEGG analysis results based on the TarBase database, the miRNAs related to lipid metabolism were retained. Finally, 22 differentially expressed miRNAs were obtained for the ceRNA network. Focusing on these miRNAs, the differential mRNAs and differential lncRNAs targeted by them were predicted under the conditions of TargetScan score ≥ 90, the Tot Score of miRanda > 140 and Tot Energy < − 20. Since mRNAs and lncRNAs often have the same expression trend in ceRNA networks, the differential lncRNA‒mRNA relationship pairs with a correlation coefficient > 0.65 and a *p* value < 0.05 were selected, and a lncRNA‒miRNA‒mRNA ceRNA network was finally constructed. The ceRNA network had a total of 28 relationship pairs (Fig. 7F).

There were a total of 10 differentially expressed miRNAs in the ceRNA network. The functional analysis of the differentially expressed mRNAs unique to youth targeted by these miRNAs revealed that these genes were enriched in fatty acid degradation as well as glycine, serine and threonine metabolism. *ACOX1* was included in the five-hub metabolism-related genes previously screened (Fig. 7G). Finally, we verified the differential expression of *CDC42SE1* and miR-378a-5p in another HBV-HCC clinical samples (Fig. 7H).

## Discussion

Compared with elderly patients, AYA HCC patients tended to have a later tumor progression and a higher degree of malignancy in previous studies [24–26]. This study focused on HBV-related liver cancer because HBV infection is the main cause of HCC in China. To highlight the effect of age, we screened patients younger than 40 years old and older than 65 years old to compare the mid- and long-term OS and tumor RFS up to five years. Similar to the results of previous studies, the OS and RFS of the AYA group were significantly worse. Moreover, youth was an independent risk factor for both OS and RFS. These results of HBV-related HCC were basically consistent with the AYA HCC from various causes.

Basic research related to AYA HCC is extremely scarce, especially on HBV-AYA HCC. Celina Ang et al. [10] discovered differentially expressed proteins such as MRP1, androgen receptor, and SPARC between AYA and elderly groups. Meanwhile, it was also found that there were significant differences between p-glycoprotein and ITOP2A in the primary tumor and secondary tumor in AYA patients. These molecules, therefore, have the potential to be biomarkers for AYA HCC. A related study analyzed the frequency of *UGT2B28* gene mutation in patients with HCC with clinical and pathological indicators [27] and found that age was an independent correlative factor for this gene mutation. *UGT2B28* is a gene related to the metabolism of bile acids and sex hormones. Therefore, metabolism was a good breakthrough point to explore the molecular features and mechanisms of differential prognosis between AYA and elderly HCC patients.

Based on the whole transcriptome sequencing results, HBV-AYA HCC-specific genes were clustered and functionally analyzed and were mainly enriched in two aspects: protein translation and processing dominated by upregulated genes and the other part was metabolic pathways with downregulated genes. It has been proven that the subsequent folding and processing of proteins is related to the occurrence and progression of tumors. The accumulation of misfolded or unfolded proteins in the ER causes ER stress and then triggers the unfolded protein response (UPR), which finally activates subsequent pathways causing cytoskeletal movement, DNA damage, and others, thereby promoting tumorigenesis and tumor migration [28, 29]. Increasing evidence has indicated that changes in the intracellular metabolic state, pH, oxygen content and transcription and translation rates may lead to ER stress. Conversely, ER stress and UPR can result in many abnormalities, including lipid metabolism, amino acid metabolism, inflammatory response, and apoptosis [30, 31].

It is widely believed that abnormalities in various metabolic processes will have an impact on the occurrence and development of tumors [32]. To explore the dominant metabolic pathway in HBV-AYA HCC, age-related GSEA was performed, and then a corresponding MPI network was constructed. Abnormal lipid metabolism, especially fatty acid metabolism had a great impact on HBV-AYA HCC. In recent years, a large amount of attention has been given to the role of fatty acid metabolism in tumorigenesis, which can provide the necessary raw material to synthesize structural lipids for actively proliferating tumor cells [33]. The induction of lipid synthesis must be closely related to cell growth and is a prerequisite for cell division. In addition, fatty acids can also affect cell transformation, tumor development and migration by modifying signalling molecules, acting as secondary messengers or ligands for autocrine receptor signalling, changing the fluidity of cell membranes, and activating tumor-related pathways [34, 35].

In this study, we also screened the unique differentially expressed lncRNAs and miRNAs of the AYA group. Through the functional analysis of target genes from multiple aspects, it was found that the metabolism-related pathways dominated by lipid metabolism were significantly enriched. Then, 22 miRNAs unique to HBV-AYA HCC were screened to construct ceRNA networks by predicted target lncRNAs and mRNAs. The differentially expressed target genes in the network were also enriched in peroxisomes, fatty acid metabolism, carbon metabolism and others.

Additionally, evidence also came from a related study on TCGA-LIHC samples grouped by five hub metabolism-related genes screened by MPIs and PPIs for differential analysis. Among the metabolism-related pathways, fatty acid elongation was at the core. Among the genes with more obvious fold changes, such as *PPT1*, *MECR*, *HADHA*, and *HADHB*, it was found that these downregulated genes were related to the synthesis of medium- and short-chain fatty acids in mitochondria that related to the degradation program. Downregulation of these genes inhibited the breakdown and oxidation of fatty acids to ensure lipid synthesis, and promoted tumor proliferation, growth, and migration [36]. Many studies have also shown that abnormal fatty acid metabolism can regulate tumor progression by affecting the NF-κB pathway, including promoting tumor proliferation, enhancing tumor invasiveness, and promoting inflammatory responses [37, 38]. Immune infiltration analysis was performed on the two groups. Interestingly, the C1 group with downregulated metabolic pathways showed increased immune infiltration. The increase in protumor indicators was more significant than that of antitumor factors. This may be related to the proinflammatory effect caused by the upregulation of the NF-κB pathway. Some studies have also reported that unsaturated fatty acids, such as arachidonic acid can activate these immune regulators [39, 40]. In addition, the increase in checkpoint inhibition and tumor-specific MHC-II may favor the effect of immune checkpoint inhibitor therapy on HCC with abnormal fatty acid metabolism [41, 42]. Therefore, the differentially expressed metabolic genes may be the reason for the different outcomes between HBV-AYA HCC patients and old HBV-HCC patients.

Frankly, due to the rarity of HBV-AYA samples and the high cost of whole transcriptome sequencing, a large number of samples are hardly involved to explore the molecular features of HBV-AYA HCC patients. Although part of our findings were confirmed in the big cohort (TCGA-LIHC), differences really exist in our cohort and the public database, for example the most downregulated genes were related to the medium- and short-chain fatty acids elongation in mitochondria in preparation for the degradation program in TCGA-LIHC samples, while the fatty acid metabolism-related genes in our cohort were more broadly enriched in the entire process of fatty acid degradation. Therefore, the findings in our study need further validation. More importantly, further basic experiments should be carried out to explore the detailed impact of fatty acid metabolism on AYA HCC development. In conclusion, according to our results, genes related to metabolism are significantly altered in the livers of HBV-AYA HCC patients, resulting in abnormal lipid metabolism, especially fatty acid metabolism. The degradation and oxidation of fatty acids are weakened, so that more fatty acids are synthesized to promote cell growth, proliferation, and metastasis. Meanwhile, abnormal fatty acid metabolism can also activate the NF-κB pathway and alter the TME, which further facilitates tumor progression. This may be the reason why HBV-AYA HCC patients have a worse prognosis and are more prone to recurrence.

## Supplementary Information

**Additional file 1**. Supplementary methods.

**Additional file 2**. Table S1. Clinical background information of HCC patients for sequencing.

**Additional file 3**. Table S2. Baseline characteristics of young and old groups

**Additional file 4**. Table S3. Primer sequences of the part of ceRNAs

**Additional file 5**. **Fig. S1**. Unique differential lncRNAs and miRNAs of AYA HCC patients. A Volcano plot of differentially expressed lncRNAs in the AYA group; B Volcano plot of differentially expressed lncRNAs in the elderly group; C Venn diagram of differentially expressed lncRNAs in the AYA and elderly group; D Volcano plot of differentially expressed miRNAs in the AYA group; E Volcano plot of differentially expressed miRNAs in the elderly group; F Venn diagram of differentially expressed miRNAs in the AYA and elderly group.

## Data Availability

The datasets used and/or analyzed during the current study are available from the corresponding author on reasonable request.

## References

[1] Sung H, Ferlay J, Siegel RL, Laversanne M, Soerjomataram I, Jemal A (2021). Global Cancer Statistics 2020: GLOBOCAN Estimates of Incidence and Mortality Worldwide for 36 Cancers in 185 Countries. CA Cancer J Clin..

[2] Chen CH, Chang TT, Cheng KS, Su WW, Yang SS, Lin HH (2006). Do young hepatocellular carcinoma patients have worse prognosis? The paradox of age as a prognostic factor in the survival of hepatocellular carcinoma patients. Liver Int Off J Int Assoc Stud Liver.

[3] Cho SJ, Yoon JH, Hwang SS, Lee HS (2007). Do young hepatocellular carcinoma patients with relatively good liver function have poorer outcomes than elderly patients?. J Gastroenterol Hepatol.

[4] Lam CM, Chan AO, Ho P, Ng IO, Lo CM, Liu CL (2004). Different presentation of hepatitis B-related hepatocellular carcinoma in a cohort of 1863 young and old patients - implications for screening. Aliment Pharmacol Ther.

[5] Li L, Xu L, Wen T, Wu H, Wang W, Yang J (2020). Poor prognoses of young hepatocellular carcinoma patients with microvascular invasion: a propensity score matching cohort study. Gastroenterol Res Pract.

[6] Diao YK, Liu JW, Wu H, Wang MD, Fan XP, Chen TH (2021). Long-term oncologic outcomes of liver resection for hepatocellular carcinoma in adolescents and young adults: a multicenter study from a hepatitis B virus-endemic area. Am J Surg.

[7] Mirici-Cappa F, Gramenzi A, Santi V, Zambruni A, Di Micoli A, Frigerio M (2010). Treatments for hepatocellular carcinoma in elderly patients are as effective as in younger patients: a 20-year multicentre experience. Gut.

[8] Zhang W, Liu C, Tan Y, Jiang L, Yan L, Yang J (2018). Role of liver resection in treating intermediate and advanced stage adolescent and young adult hepatocellular carcinoma patients: a propensity-matching cohort study. Int J Surg.

[9] Au KY, Chan KK, Lo RC (2021). A clinicopathological study of young-onset hepatocellular carcinoma. Anticancer Res.

[10] Ang C, Shields A, Xiu J, Gatalica Z, Reddy S, Salem ME (2017). Molecular characteristics of hepatocellular carcinomas from different age groups. Oncotarget.

[11] Subramanian A, Tamayo P, Mootha VK, Mukherjee S, Ebert BL, Gillette MA (2005). Gene set enrichment analysis: a knowledge-based approach for interpreting genome-wide expression profiles. Proc Natl Acad Sci USA.

[12] Ghazalpour A, Doss S, Zhang B, Wang S, Plaisier C, Castellanos R (2006). Integrating genetic and network analysis to characterize genes related to mouse weight. PLoS Gen.

[13] von Mering C, Jensen LJ, Snel B, Hooper SD, Krupp M, Foglierini M (2005). STRING: known and predicted protein-protein associations, integrated and transferred across organisms. Nucleic Acids Res.

[14] Chen D, Zhang Y, Wang W, Chen H, Ling T, Yang R (2021). Identification and characterization of robust hepatocellular carcinoma prognostic subtypes based on an integrative metabolite-protein interaction network. Adv Sci.

[15] Karreth FA, Pandolfi PP (2013). ceRNA cross-talk in cancer: when ce-bling rivalries go awry. Cancer Discover.

[16] Goldman MJ, Craft B, Hastie M, Repečka K, McDade F, Kamath A (2020). Visualizing and interpreting cancer genomics data via the Xena platform. Nat Biotechnol.

[17] Yoshihara K, Shahmoradgoli M, Martínez E, Vegesna R, Kim H, Torres-Garcia W (2013). Inferring tumour purity and stromal and immune cell admixture from expression data. Nat Commun.

[18] Newman AM, Liu CL, Green MR, Gentles AJ, Feng W, Xu Y (2015). Robust enumeration of cell subsets from tissue expression profiles. Nat Methods.

[19] Bagaev A, Kotlov N, Nomie K, Svekolkin V, Gafurov A, Isaeva O (2021). Conserved pan-cancer microenvironment subtypes predict response to immunotherapy. Cancer Cell.

[20] Chin CH, Chen SH, Wu HH, Ho CW, Ko MT, Lin CY (2014). cytoHubba: identifying hub objects and sub-networks from complex interactome. BMC systems biology..

[21] Reina M, Espel E (2017). Role of LFA-1 and ICAM-1 in Cancer. Cancers.

[22] Quail DF, Joyce JA (2013). Microenvironmental regulation of tumor progression and metastasis. Nat Med.

[23] Shackelford DB, Shaw RJ (2009). The LKB1-AMPK pathway: metabolism and growth control in tumour suppression. Nat Rev Cancer.

[24] Shen J, Li C, Yan L, Li B, Xu M, Yang J (2018). Short- and long-term outcomes between young and older HCC Patients exceeding the milan criteria after hepatectomy. Ann Hepatol.

[25] Lee JS, Kim JM, Lee S, Choi JY, Cho W, Choi GS (2015). The prognosis in cases of hepatocellular carcinoma after hepatectomy: young patients versus older patients. Korean J Hepatobiliary Pancreat Surg.

[26] Ng KKC, Cheng NMY, Huang J, Liao M, Chong CCN, Lee KF (2021). Development and validation of a novel nomogram predicting 10-year actual survival after curative hepatectomy for hepatocellular carcinoma. The Surgeon J Royal Colleges Surgeons Edinburgh Ireland.

[27] Le PH, Kuo CJ, Hsieh YC, Chen TH, Lin CL, Yeh CT (2019). Ages of hepatocellular carcinoma occurrence and life expectancy are associated with a UGT2B28 genomic variation. BMC Cancer.

[28] Hetz C, Zhang K, Kaufman RJ (2020). Mechanisms, regulation and functions of the unfolded protein response. Nat Rev Mol Cell Biol.

[29] Ojha R, Amaravadi RK (2017). Targeting the unfolded protein response in cancer. Pharmacol Res.

[30] Chen X, Cubillos-Ruiz JR (2021). Endoplasmic reticulum stress signals in the tumour and its microenvironment. Nat Rev Cancer.

[31] Moncan M, Mnich K, Blomme A, Almanza A, Samali A, Gorman AM (2021). Regulation of lipid metabolism by the unfolded protein response. J Cell Mol Med.

[32] Vander Heiden MG, DeBerardinis RJ (2017). Understanding the Intersections between metabolism and cancer biology. Cell.

[33] Currie E, Schulze A, Zechner R, Walther TC, Farese RV (2013). Cellular fatty acid metabolism and cancer. Cell Metab.

[34] Röhrig F, Schulze A (2016). The multifaceted roles of fatty acid synthesis in cancer. Nat Rev Cancer.

[35] Carracedo A, Cantley LC, Pandolfi PP (2013). Cancer metabolism: fatty acid oxidation in the limelight. Nat Rev Cancer.

[36] Riezman H (2007). The long and short of fatty acid synthesis. Cell.

[37] Senga S, Kobayashi N, Kawaguchi K, Ando A, Fujii H (2018). Fatty acid-binding protein 5 (FABP5) promotes lipolysis of lipid droplets, de novo fatty acid (FA) synthesis and activation of nuclear factor-kappa B (NF-κB) signaling in cancer cells. Biochim Biophys Acta.

[38] Keshk WA, Zineldeen DH, Wasfy RE, El-Khadrawy OH (2014). Fatty acid synthase/oxidized low-density lipoprotein as metabolic oncogenes linking obesity to colon cancer via NF-kappa B in Egyptians. Med Oncol.

[39] Zhang H, Liu Y, Weng J, Usuda K, Fujii K, Watanabe G (2017). Decrease of lactogenic hormones induce epithelial-mesenchymal transition via TGFβ1 and arachidonic acid during mammary gland involution. J Reprod Dev.

[40] Harbige LS, Layward L, Morris-Downes MM, Dumonde DC, Amor S (2000). The protective effects of omega-6 fatty acids in experimental autoimmune encephalomyelitis (EAE) in relation to transforming growth factor-beta 1 (TGF-beta1) up-regulation and increased prostaglandin E2 (PGE2) production. Clin Exp Immunol.

[41] Barrueto L, Caminero F, Cash L, Makris C, Lamichhane P, Deshmukh RR (2020). Resistance to checkpoint inhibition in cancer immunotherapy. Trans Oncol.

[42] Bagchi S, Yuan R, Engleman EG (2021). Immune checkpoint inhibitors for the treatment of cancer: clinical impact and mechanisms of response and resistance. Annu Rev Pathol.

