# Supplementary material for: Clinical prognosis and related molecular features of hepatitis B-associated adolescent and young adult hepatocellular carcinoma
Source: Hum Genomics. 2023 Jun 13;17:52. doi: 10.1186/s40246-023-00500-9 (PMC10262462; doi:10.1186/s40246-023-00500-9)
Supplement: Supplementary file 1 — Additional file 1. Supplementary methods. [file 40246_2023_500_MOESM1_ESM.docx]

**SUPPLEMENTARY MATERIAL**

**Supplementary methods**

**Detailed clinical study design**

All HCC patients included in this study were HBV infected, and finally 289 HBV-related AYA HCC patients and 257 HBV-related elderly HCC patients who underwent hepatectomy in West China Hospital, Sichuan University, Chengdu, China from 2010 to 2014, were included. The data of all patients were retrospectively reviewed. Inclusion criteria included the age of patients (AYA group 15-40 years old, elderly group over 60 years old), no history of previous anticancer therapy, radical resection of liver tumours and the histopathologically proven HCC, grade A or B Child-Pugh liver function. Exclusion criteria included extrahepatic metastasis of primary liver cancer, simultaneously underwent resection and radiofrequency ablation and palliative resection and targeted therapy, the mixed or other type of liver cancer, grade C Child-Pugh liver function, pregnant or breastfeeding women, patients with a history of mental illness or psychotropic substance abuse and HIV-infected people.

Laboratory blood examination, including routine blood test, liver and renal function, coagulation tests, tumour markers like alpha-fetoprotein (AFP) and infectious disease screening were completely tested. Preoperative chest X-ray, electrocardiogram and enhanced CT or MRI of abdomen to exclude the cardiopulmonary diseases and confirm the HCC diagnosis. Postoperative HCC diagnosis was confirmed by histopathological examination. Two experienced pathologists confirmed the final diagnosis and all specimens were routinely examined for the presence of the microvascular invasion (MVI). Liver and renal function test, serum AFP assay, routine blood test and abdominal ultrasonography were performed every 3 months in the first and second postoperative year and every 6 months in the subsequent years. Patients who survived and were not lost to follow-up were followed up to at least 5 years. Overall survival (OS) and recurrence-free survival (RFS) were the endpoints of our study. OS was calculated from the date of radical liver resection to the date of patient’s death or the date of last follow-up visit. DFS was measured from the date of radical liver resection to the date when tumour recurrence was diagnosed.

**Collection of HCC specimens and sequencing**

The sequencing work of this study was completed by LC-Bio Technologies (Hangzhou) Co., Ltd. Total RNA was isolated and purified using TRIzol reagent (Invitrogen, Carlsbad, CA, USA) following the manufacturer's procedure. After removing ribosomal RNAs, the remaining RNAs were fragmented into small pieces, and the RNA library was constructed. Finally, we performed high-throughput sequencing following the vendor's recommended protocol as well as data mapping, analysis, and annotation..

**Screening of AYA unique different mRNAs, miRNAs and lncRNAs**

Differential expression analysis of mRNAs and lncRNAs in the AYA group and elderly group with a p value <0.05 and |log2-fold change (FC)|>1 threshold was performed by the “edgeR” package in R. Then, unique differential mRNAs and lncRNAs of the AYA group were selected by Venn diagram (https://bioinfogp.cnb.csic.es/tools/venny) with an adjusted p value <0.05 and |log2-fold change (FC)|>1 threshold. For AYA unique differential mRNAs, the mean fragments per kilobase of transcript per million mapped reads (FPKM) >15 was an additional condition. The expression levels of miRNAs were normalized following the procedures described in a previous study[1] with minor modifications. Then, a t test of the two groups of samples with biological replicates was performed on the expression of miRNAs between the tumour tissue and adjacent tissue of the AYA group and the elderly group with a p value <0.05 and |log2-fold change (FC)|>1 threshold. The unique differential miRNAs of the AYA group were selected by Venn diagram with p value <0.01. Heatmaps were plotted by https://www.bioinformatics.com.cn.

**Functional enrichment analysis**

For the differentially expressed mRNAs in AYA HCC, GO and KEGG functional analysis was performed using the “Cluster Profiler” package in R. Gene set enrichment analysis (GSEA) was also performed by the “Cluster Profiler” package. The age-related GSEA was using the “fgsea” package, in which the AYA unique differentially expressed genes arranged by correlation between age and gene expression. The KEGG enrichment analysis of differential miRNAs based on the TarBase database was performed through the mirPath function in DIANA TOOLS (http://snf-515788.vm.okeanos.grnet.gr/).

**Mfuzz clustering analysis**

According to the corresponding age of the HBV-HCC patients included for sequencing, the tumour samples were divided into 4 groups (<25, 25-30, 65-70, >70 years old). Mfuzz clustering analysis was performed using “Mfuzz” package in R, and the genes were set into 5 groups. Further functional analysis was performed on the differential gene clusters.

**WGCNA analysis**

The expression of unique differential genes of AYA and elderly liver tumour samples were used as input matrix. Clinical indicators such as age, tumour recurrence within 1 year, height, weight, AFP, abnormal prothrombin, total tumour diameter, microvascular invasion, pathological grade, TMN grade, etc. of the corresponding HCC patients were collected. The ”WGCNA” package in R was used for analysis. Outlier samples were removed by drawing a clustering tree and set a soft threshold of 6.

**MPI and PPI Network Construction**

PPIs were obtained from STRING (https://cn.string-db.org/) and optimized the network by Cytoscape. The hub genes were screened out by the MCC algorithm in Cyto-Hubba. The MPI network was constructed as previous described, by which MPIs extracted from four resources including KEGG, Reactome, Human-GEM, and BRENDA. Genes in MPIs were ranged by degree using cytoNCA in Cytoscape. Combining PPIs and MPIs, metabolism-related hub genes were screened out from the intersection of key genes of two networks.

**Establishment of the ceRNA network**

The miRNAs are known to mainly cause gene silence by binding to downstream target mRNA at the post-transcriptional level, while lncRNAs can act as baits for miRNA binding and counteract the repressive activity of miRNA, which is the fundamental basis of ceRNA network.[1] lncRNA-mRNA co-expression relationship and the differential miRNA-mRNA and the differential miRNA-lncRNA regulatory relationships were integrated to construct the ceRNA network. Context score percentile>90 by TargetScan, Tot Score>140 and Tot Energy<-20 by miRanda were filters for target ceRNAs. Pearson correlation coefficient>0.65 and p value <0.05 were the threshold of lncRNA-mRNA co-expression relationship.

**TCGA data preparation and processing**

Genomic Data Commons (GDC) TCGA LIHC (liver hepatocellular carcinoma) data provided by the University of California Santa Cruz (UCSC) Xena database (https://xenabrowser.net/datapages/) was used in this study for further validation. PCA analysis was performed on the HCC samples in the TCGA database based on the expression of metabolic-related hub genes. Samples were divided into two groups with PC1=PC2 as the dividing line. C1 group was the part of PC1>PC2, and C2 group was the part of PC1<PC2. The gene difference analysis of the two groups was further carried out through the “DESeq2” package in R. The threshold was set as p value <0.01 and |log2 fold change (FC)|>2. Functional analysis was performed by “Cluster Profiler” package and PPIs of key pathways was established using STRING with outcomes of GSEA analysis. The Kaplan–Meier method was used for prognosis analysis.

**Immune infiltrate levels and expression analysis**

The immune score and stromal score of C1 and C2 groups were calculated by the “estimate” package in R[2]. The proportion of the 22 types of immune cells in the tumour microenvironment (TME) of each sample was evaluated using the “CIBERSORT” package in R[3]. A list of 29 functional gene expression signatures (Fges) representing cellular and functional TME properties was proposed by Bagaev et al[4]. The expression of 29-Fges related genes was integrated and compared between two groups using the ssGSEA algorithm via the “GSVA” package in R.

**RT-qPCR assay**

The mRNAs were reversely transcribed using RT Master Mix for qPCR (MCE, New Jersey, USA), while miRNA was reverse transcribed by stem-loop method. Taq Pro Universal SYBR qPCR Master Mix (Vazyme, Nanjing, China) was used for qRT-PCR. The lncRNA and mRNA levels were normalized by GAPDH. The miRNA level was normalized by small nuclear U6. The relative expression levels were determined by the 2^−ΔCt^ or 2^−ΔΔCt^ method. Primers were listed in Supplementary Table 3.

**Reference**

1. Karreth FA, Pandolfi PP. ceRNA cross-talk in cancer: when ce-bling rivalries go awry. Cancer discovery. 2013;3(10):1113-21. doi: 10.1158/2159-8290.Cd-13-0202.

2. Yoshihara K, Shahmoradgoli M, Martínez E, Vegesna R, Kim H, Torres-Garcia W, et al. Inferring tumour purity and stromal and immune cell admixture from expression data. Nature communications. 2013;4:2612. doi: 10.1038/ncomms3612.

3. Newman AM, Liu CL, Green MR, Gentles AJ, Feng W, Xu Y, et al. Robust enumeration of cell subsets from tissue expression profiles. Nature methods. 2015;12(5):453-7. doi: 10.1038/nmeth.3337.

4. Bagaev A, Kotlov N, Nomie K, Svekolkin V, Gafurov A, Isaeva O, et al. Conserved pan-cancer microenvironment subtypes predict response to immunotherapy. Cancer cell. 2021;39(6):845-65.e7. doi: 10.1016/j.ccell.2021.04.014.
