# Supplementary material for: Clinical prognosis and related molecular features of hepatitis B-associated adolescent and young adult hepatocellular carcinoma
Source: Hum Genomics. 2023 Jun 13;17:52. doi: 10.1186/s40246-023-00500-9 (PMC10262462; doi:10.1186/s40246-023-00500-9)
Supplement: Supplementary file 2 — Additional file 2. Table S1. Clinical background information of HCC patients for sequencing. [file 40246_2023_500_MOESM2_ESM.docx]

**Supplementary Table S1 Clinical background information of HCC patients for sequencing**

|  | **age** | **R1** | **height**  **(cm)** | **weight**  **(kg)** | **AFP**  **(ng/ml)** | **Pivka-II**  **(mAU/ml)** | **TTS**  **(cm)** | **MVI** | **grade** | **Tstage** |
| --- | --- | --- | --- | --- | --- | --- | --- | --- | --- | --- |
| YT1 | 22 | 0 | 171 | 71 | 179 | 452 | 4.3 | 0 | 2 | 1 |
| YT11 | 29 | 1 | 172 | 78 | 1210 | 36 | 3.7 | 1 | 3 | 2 |
| YT12 | 28 | 1 | 165 | 54 | 26.4 | 4938 | 5.2 | 1 | 3 | 2 |
| YT3 | 24 | 1 | 158 | 50 | 1210 | 2739 | 8.8 | 1 | 1 | 2 |
| YT4 | 28 | 0 | 170 | 68 | 10.7 | 5180 | 7.2 | 1 | 1 | 2 |
| YT5 | 24 | 1 | 163 | 54 | 1210 | 5462 | 14.1 | 1 | 1 | 3 |
| YT6 | 31 | 1 | 165 | 61 | 1210 | 3196 | 12 | 1 | 1 | 2 |
| YT7 | 30 | 0 | 171 | 66 | 1210 | 23687 | 13.2 | 1 | 1 | 3 |
| YT8 | 29 | 1 | 173 | 70 | 1210 | 189 | 6 | 1 | 1 | 2 |
| YT9 | 31 | 0 | 170 | 62 | 1210 | 19 | 3.2 | 0 | 3 | 1 |
| OT1 | 68 | 0 | 168 | 52 | 1210 | 15649 | 7.5 | 1 | 1 | 2 |
| OT11 | 72 | 1 | 162 | 53 | 1210 | 44244 | 19 | 1 | 3 | 3 |
| OT12 | 64 | 0 | 178 | 79 | 18.1 | 1245 | 6.5 | 0 | 3 | 1 |
| OT2 | 69 | 0 | 165 | 50.5 | 91.1 | 3190 | 5 | 0 | 1 | 1 |
| OT4 | 76 | 0 | 167 | 70 | 75.2 | 36 | 6.3 | 0 | 3 | 2 |
| OT5 | 67 | 1 | 158 | 56 | 1210 | 13372 | 12.5 | 1 | 1 | 4 |
| OT6 | 68 | 0 | 170 | 69.5 | 1210 | 15082 | 5.5 | 1 | 2 | 2 |
| OT7 | 65 | 0 | 162 | 73 | 208 | 163 | 3.7 | 0 | 3 | 1 |
| OT8 | 67 | 0 | 161 | 61 | 1210 | 72 | 4.2 | 1 | 3 | 2 |
| OT9 | 66 | 0 | 158 | 47 | 3.68 | 21303 | 17.2 | 1 | 3 | 3 |

**Abbreviation: YT:** tumor of AYA group; **OT:** tumor of elderly group; **R1:** recurrence within 1 year; **AFP:** alphafetoprotein;**Pivka-II:**Protein Induced by Vitamin K Absence or Antagonist-II**; TTS:** total tumor size; **MVI:** microvascular invasion; **0:** none; **1:** exist. For differentiation: **1:** mediate differentiation; **2:** mediate-low differentiation; **3:** low differentiation; For T stage: **1:** T1; **2:** T2; **3:** T3; **4:** T4.
