# Supplementary material for: Clinical prognosis and related molecular features of hepatitis B-associated adolescent and young adult hepatocellular carcinoma
Source: Hum Genomics. 2023 Jun 13;17:52. doi: 10.1186/s40246-023-00500-9 (PMC10262462; doi:10.1186/s40246-023-00500-9)
Supplement: Supplementary file 3 — Additional file 3. Table S2. Baseline characteristics of young and old groups [file 40246_2023_500_MOESM3_ESM.docx]

**Supplementary Table S2**  **Baseline characteristics of young and old groups**

|  | **Old group**  **(n=257)** | **Young group**  **(n=289)** | ***P*** |
| --- | --- | --- | --- |
| **Male (%)** | 207 (80.5) | 237 (82.0) | 0.662 |
| **B stage liver function(%)** | 31 (12.1) | 26 (9.0) | 0.264 |
| **BMI (%)** |  |  | 0.911 |
| **<18.5** | 16 (6.2) | 20 (6.9) |  |
| **18.5≤BMI<24** | 186 (72.4) | 210 (72.7) |  |
| **≥24** | 55 (21.4) | 59 (20.4) |  |
| **Hb (mean (SD),g/L)** | 131.5 (17.4) | 141.0 (21.3) | <0.001 |
| **PLT (mean (SD),10^9/L)** | 140.1 (67.3) | 166.8 (78.9) | <0.001 |
| **NLR (median [IQR])** | 2.4 [1.8, 4.0] | 2.7 [1.8, 3.8] | 0.639 |
| **TB (median [IQR],μmol/L)** | 14.6 [10.8, 18.9] | 13.5 [10.0, 18.3] | 0.073 |
| **DB (median [IQR],μmol/L)** | 5.4 [3.9, 7.4] | 5.1 [3.7, 7.0] | 0.404 |
| **AST (median [IQR],U/L)** | 41.0 [28.0, 62.0] | 42.0 [31.0, 65.0] | 0.158 |
| **ALT (median [IQR],U/L)** | 34.0 [23.0, 57.0] | 45.0 [31.0, 68.2] | <0.001 |
| **ALB (median [IQR],g/L)** | 39.8 [36.3, 43.0] | 41.6 [38.1, 45.0] | <0.001 |
| **PT (mean (SD),s)** | 11.9 (1.3) | 12.1 (1.5) | 0.093 |
| **AFP≥400ng/mL (%)** | 73 (28.4) | 174 (60.2) | <0.001 |
| **Region (%)** | |  | 0.293 |
| **Left lobe** | 70 (27.2) | 62 (21.5) |  |
| **Middle lobe/Both lobes** | 28 (10.9) | 36 (12.5) |  |
| **Right lobe** | 159 (61.9) | 191 (66.1) |  |
| **Multiple tumors (%)** | 37 (14.4) | 52 (18.0) | 0.296 |
| **Max tumor size >5cm (%)** | 130 (50.6) | 168 (58.1) | 0.085 |
| **MVI(+) (%)** | 58 (22.6) | 119 (41.2) | <0.001 |
| **Low differentiated tumor (%)** | 100 (38.9) | 124 (42.9) | 0.384 |

**Abbreviation: BMI:** body mass index; **Hb:** hemoglobin; **PLT:** platelet; **NLR:** neutrophil-to-lymphocyte ratio; **TB:** total bilirubin; **DB:** direct bilirubin; **AST:** aspartate aminotransferase; **ALT:** alanine aminotransferase; **ALB:** albumin; **PT:** prothrombin time; **AFP:** alphafetoprotein; **MVI:** microvascular invasion.
