# Supplementary material for: Clinical prognosis and related molecular features of hepatitis B-associated adolescent and young adult hepatocellular carcinoma
Source: Hum Genomics. 2023 Jun 13;17:52. doi: 10.1186/s40246-023-00500-9 (PMC10262462; doi:10.1186/s40246-023-00500-9)
Supplement: Supplementary file 4 — Additional file 4. Table S3. Primer sequences of the part of ceRNAs [file 40246_2023_500_MOESM4_ESM.docx]

**Supplementary Table S3**  **Primer sequences of the part of ceRNAs**

| **Primer name** | **Sequence (5’-3’)** |
| --- | --- |
| CDC42SE1-F | GGATCTCAAGGTCAGGGTTCCG |
| CDC42SE1-R | GAACAGGACCAGAGAGAGAGGT |
| GAPDH-F | GGAGCGAGATCCCTCCAAAAT |
| GAPDH-R | GGCTGTTGTCATACTTCTCATGG |
| hsa-miR-378a-5p-J | GTCGTATCCAGTGCAGGGTCCGAGGTATTCGCACTGGATACGACACACAG |
| hsa-miR-378a-5p-F | GCTCCTGACTCCAGGTC |
| hsa-miR-378a-5p-R | GCAGGGTCCGAGGTATTC |
| U6-F | CTCGCTTCGGCAGCACA |
| U6-R | AACGCTTCACGAATTTGCGT |

**Abbreviation: F:** forward primer; **R:** reverse primer; **J:** stem-loop primer.
