# Supplementary figures and images for: Clinical prognosis and related molecular features of hepatitis B-associated adolescent and young adult hepatocellular carcinoma
Source: Hum Genomics. 2023 Jun 13;17:52. doi: 10.1186/s40246-023-00500-9 (PMC10262462; doi:10.1186/s40246-023-00500-9)

**A**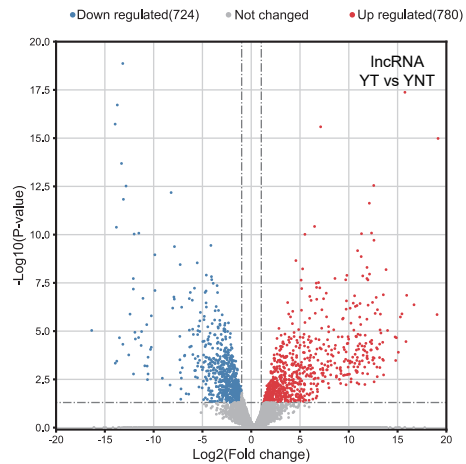**B**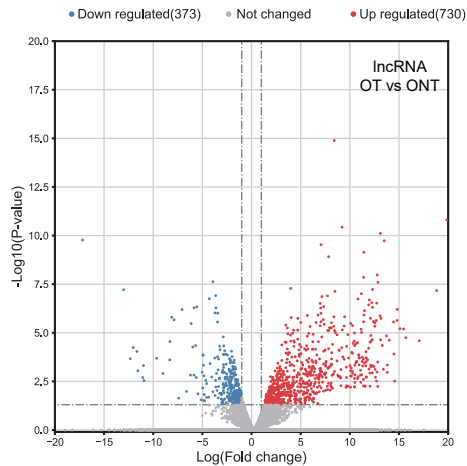**C**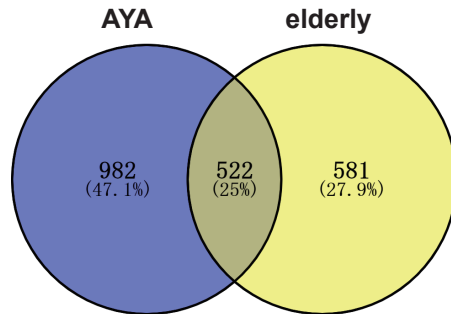**D**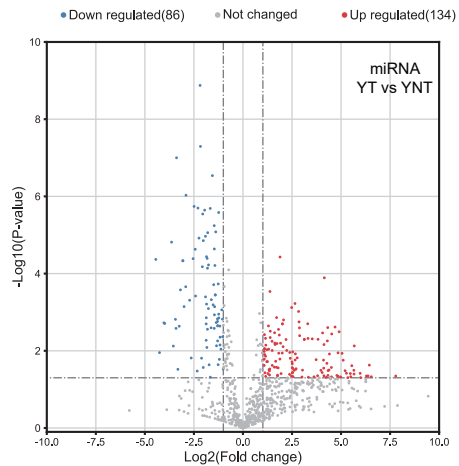**E**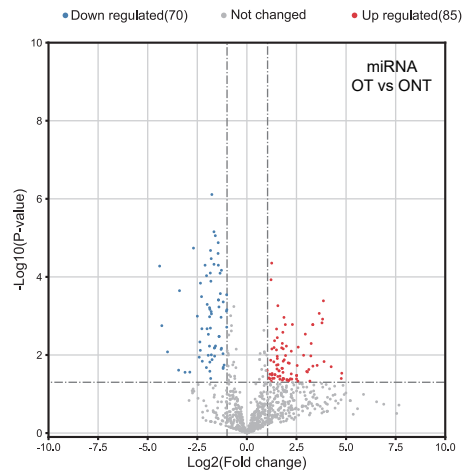**F**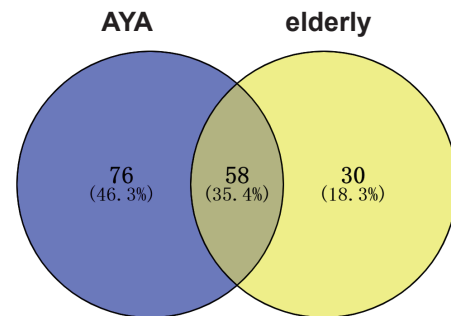

Supplement: Supplementary file 5 — Additional file 5. Fig. S1. Unique differential lncRNAs and miRNAs of AYA HCC patients. A Volcano plot of differentially expressed lncRNAs in the AYA group; B Volcano plot of differentially expressed lncRNAs in the elderly group; C Venn diagram of differentially expressed lncRNAs in the AYA and elderly group; D Volcano plot of differentially expressed miRNAs in the AYA group; E Volcano plot of differentially expressed miRNAs in the elderly group; F Venn diagram of differentially expressed miRNAs in the AYA and elderly group. [file 40246_2023_500_MOESM5_ESM.pdf]
